# Resistant starch lowers postprandial glucose and leptin in overweight adults consuming a moderate-to-high-fat diet: a randomized-controlled trial

**DOI:** 10.1186/s12937-017-0235-8

**Published:** 2017-02-21

**Authors:** Mindy Patterson Maziarz, Sara Preisendanz, Shanil Juma, Victorine Imrhan, Chandan Prasad, Parakat Vijayagopal

**Affiliations:** 1 Department of Nutrition and Food Sciences, Institute of Health Sciences, Texas Woman’s University, 6700 Fannin Street, Houston, TX 77030 USA; 2 Department of Nutrition and Food Sciences, Texas Woman’s University, P.O Box 425888, Denton, TX 76204 USA; 3 Department of Medicine (Endocrinology), Louisiana State University Health Science Center, New Orleans, LA 70112 USA

**Keywords:** Resistant starch, Satiety, Overweight, Gut peptides, Fiber, Leptin, PYY

## Abstract

**Background:**

High-amylose maize resistant starch type 2 (HAM-RS2) stimulates gut-derived satiety peptides and reduces adiposity in animals. Human studies have not supported these findings despite improvements in glucose homeostasis and insulin sensitivity after HAM-RS2 intake which can lower adiposity-related disease risk. The primary objective of this study was to evaluate the impact of HAM-RS2 consumption on blood glucose homeostasis in overweight, healthy adults. We also examined changes in biomarkers of satiety (glucagon-like peptide-1 [GLP-1], peptide YY [PYY], and leptin) and body composition determined by anthropometrics and dual-energy x-ray absorptiometry, dietary intake, and subjective satiety measured by a visual analogue scale following HAM-RS2 consumption.

**Methods:**

Using a randomized-controlled, parallel-arm, double-blind design, 18 overweight, healthy adults consumed either muffins enriched with 30 g HAM-RS2 (*n* = 11) or 0 g HAM-RS2 (control; *n* = 7) daily for 6 weeks. The HAM-RS2 and control muffins were similar in total calories and available carbohydrate.

**Results:**

At baseline, total PYY concentrations were significantly higher 120 min following the consumption of study muffins in the HAM-RS2 group than control group (*P* = 0.043). Within the HAM-RS2 group, the area under the curve (AUC) glucose (*P* = 0.028), AUC leptin (*P* = 0.022), and postprandial 120-min leptin (*P* = 0.028) decreased independent of changes in body composition or overall energy intake at the end of 6 weeks. Fasting total PYY increased (*P* = 0.033) in the HAM-RS2 group, but changes in insulin or total GLP-1 were not observed. Mean overall change in subjective satiety score did not correlate with mean AUC biomarker changes suggesting the satiety peptides did not elicit a satiation response or change in overall total caloric intake. The metabolic response from HAM-RS2 occurred despite the habitual intake of a moderate-to-high-fat diet (mean range 34.5% to 39.4% of total calories).

**Conclusion:**

Consuming 30 g HAM-RS2 daily for 6 weeks can improve glucose homeostasis, lower leptin concentrations, and increase fasting PYY in healthy overweight adults without impacting body composition and may aid in the prevention of chronic disease. However, between-group differences in biomarkers were not observed and future research is warranted before specific recommendations can be made.

**Trial registration:**

None.

## Introduction

Epidemiological observations show that consuming a diet high in fiber can lower the risk for obesity, obesity-related comorbidities, and reduce all-cause mortality [1, 2]. One systematic review that examined the effects of dietary fiber on body weight reported that a 0.4% reduction in body weight can be achieved by consuming most dietary fibers for 4 weeks [3]. However, the amount of weight lost was dependent on the physiochemical properties (solubility, fermentability, and viscosity) of each type of fiber [3].

The purported mechanisms by which fiber contributes to weight loss such as altering gut motility, attenuating nutrient absorption, and lowering overall caloric intake are also associated with the physiochemical properties [2, 4]. Fermentable fibers are receiving attention because the metabolites produced from bacterial fermentation in the gastrointestinal (GI) tract can influence body weight. These fibers produce short chain fatty acids (SCFA; acetate, propionate, butyrate) in the distal intestine that stimulate the release of glucagon-like peptide-1 (GLP-1) and peptide YY (PYY) that act synergistically with leptin, an adipokine primarily released from adipose tissue, to induce satiety and regulate energy expenditure through central nervous system actions [5–7].

Despite increased SCFA production from fiber fermentation, the relationship between GLP-1 and PYY on satiety and food intake in humans is inconsistent. After consuming a standardized breakfast on the morning immediately following 3 days of consecutive intake of a barley kernel-based bread with resistant starch, fasting plasma GLP-1 and postprandial PYY concentrations increased in healthy middle-aged adults [8]. However, changes in appetite sensations (satiety, hunger, and desire to eat) did not occur [8]. Similarly, overweight women did not elicit a postprandial subjective satiety response despite improvements of GLP-1 and PYY after consuming an enzyme-hydrolyzed arabinoxylan from wheat or intact arabinoxylan from flax at breakfast [9]. In contrast, in healthy adults, the upregulation of GLP-1 and PYY corresponded with enhanced subjective satiety immediately after resistant maltodextrin intake [10]. However, subsequent energy intake was not suppressed despite these improvements. One recent trial found improvements in the area under the curve (AUC) PYY that corresponded with satiety and a 14% reduction in food intake in healthy adults consuming 15 g unripe banana flour rich in resistant starch for 6 weeks [11]. The different satiety peptides and satiation responses may be related to fermentability patterns, the type, amount, and duration of fiber intake, and the gut microbiota composition of individuals. Also, blood gut peptides may be too low in concentration to cross the blood brain barrier or the individuals may have hypothalamic resistance, which can occur from a high-fat diet even in lieu of obesity [12].

High-amylose maize resistant starch type 2 (HAM-RS2) is an insoluble, nonviscous fermentable fiber that has been shown to improve glucose homeostasis and/or peripheral insulin sensitivity among individuals who were healthy with normal glucose homeostasis [13] or those with metabolic syndrome [14–16]. However, many of these trials of longer duration did not report or show improvements in blood concentrations of gut peptides, satiety responses, or changes in food intake. HAM-RS2 may exert its benefits on glucose metabolism by increasing SCFA in the blood to alter free fatty acid and glycerol release from adipocytes and increased fat oxidation [17], modulate bile acid metabolism [18], or alter the gut microbiota profile [19].

Most previous trials reported the impact of HAM-RS2 on glucose homeostasis in either healthy individuals or those with metabolic syndrome [12–15]. Therefore, our primary aim was to determine the impact of the daily consumption of 30 g HAM-RS2 incorporated into muffins for 6 weeks on glucose homeostasis in normoglycemic, healthy overweight adults at risk for developing glycemic abnormalities. We also measured fasting and postprandial biomarker concentrations known to influence satiety (GLP-1, PYY, and leptin), subjective satiety, dietary intake, and body composition in these individuals.

## Materials and Methods

### Participants

Healthy overweight adults with a body mass index (BMI)  ≥ 28 kg/m^2^ between 18 and 50 years of age of any race or ethnicity were recruited from Denton, Texas and the surrounding area. Participants were sedentary (<20 min of activity no more than 2 days per week) for at least 6 months prior to enrollment in the study. Exclusion criteria included those diagnosed with or taking medication(s) for chronic conditions, such as diabetes, hypertension, cancer, diseases of the liver, kidney, or heart, or other metabolic disorders. Participants were also excluded if they gained or lost a significant amount of weight or followed a special diet ≤ 3 months prior to enrollment, consumed vitamins, minerals, or antioxidants in excess of amounts found in a daily multivitamin tablet, or dietary supplements known to alter metabolism, had an intolerance to the study foods, or smoked. Women who were pregnant, lactating, or interested in becoming pregnant were not eligible. The study was approved by the Institutional Review Board at Texas Woman’s University. All participants provided written consent prior to study enrollment.

### Protocol

The study was a randomized-controlled, parallel-arm, double-blind design lasting 6 weeks. Individuals who met the screening criteria and agreed to participate in the study were randomized to either the HAM-RS2 group or control group using a random numbers generator from SPSS version 19 (IBM Corporation, Armonk, NY, USA).

Prior to participant randomization, the study muffins were formulated at the University food preparation laboratory. The treatment muffins were developed to provide 50 g Hi-Maize® 260 resistant starch (~30 g HAM-RS2, ~20 g equal mixture slowly and rapidly digestible starch; Ingredion Incorporated, Westchester, IL, USA) daily. The control muffins (0 g HAM-RS2) were developed to contain similar amounts of available carbohydrate and minimal differences in total calories than the treatment muffins (Table 1). Each 60 g treatment muffin contained 16.7 g Hi-Maize® 260 resistant starch to provide 10 g HAM-RS2, therefore three muffins (180 g cooked) were required to obtain 30 g HAM-RS2 daily as indicated in the protocol. No difference in overall muffin likeability was found based on sensory evaluations of HAM-RS2 and control muffins using a 9-point hedonic scale prior to the implementation of this study [20]. All study muffins were prepared, packaged, and labeled by culinary and nutrition students twice per week to ensure freshness and quality. Two flavors of the treatment and control muffins were developed, pumpkin spice and cranberry spice. The different flavors of muffins were administered on alternating weeks to alleviate monotony and improve compliance. The two flavors of muffins were closely matched for total calories and macronutrients. The cranberry spice muffins included 4.3 g dried cranberries, but no pumpkin puree. The pumpkin spice muffins included 6.8 g pumpkin puree without dried cranberries. Different amounts of dried cranberries and pumpkin puree were added so the caloric value of each muffin type was similar.

**Table 1:** Comparison of ﻿nutrients ﻿between muffins^a^

Nutrient	HAM-RS2	Control
Energy (kcal)^b^	349.6	389.1
Available carbohydrate (g)^c^	70.5	71.6
Protein (g)	5.4	10.6
Fat (g)	5.1	6.6
Fiber (g)	30.9	11.4

^a^Nutrient analysis based on the amount consumed daily by each participant; 180 g cooked (baked) weight. Analysis conducted by Pope Labs, Irving, Texas using the following Official AOAC Methods: moisture 925.10; oil 923.03; ash 923.03; nitrogen 988.05A; total dietary fiber 991.43

^b^Energy does not include contribution from short chain fatty acids associated with fermentation

^c^Available carbohydrate excludes fiber

The participants arrived for baseline data collection following an overnight fast. Anthropometric measurements were obtained in triplicate followed by the baseline blood collection (time 0). Participants consumed all three of their respective study muffins within 15 min along with 6 oz of orange juice. Immediately following muffin intake the participants completed a visual analogue scale (VAS). Four additional postprandial blood samples were collected at 15, 30, 60, and 120 min. Nutrition education and body composition analysis occurred between blood collections. Participants were instructed by a Registered Dietitian Nutritionist (RDN) to follow a balanced diet according to the Dietary Guidelines for Americans 2010 [21] adjusted for the caloric value of the study muffins and to remain sedentary during the study. Body composition was determined by whole body dual-energy x-ray absorptiometry (DXA) using the Lunar DPX NT model (GE Healthcare, Fairfield, CT, USA). Body composition analysis and blood collections were repeated using the same protocol as baseline at the end of the intervention (week 6).

### Dietary intake and bowel habits

Three days prior to baseline measurements, at midpoint (week 3), and the end of the study (week 6) all participants completed bowel habit logs and dietary intake journals for 3 days as instructed by a RDN. Dietary intake was analyzed using the United States Department of Agriculture National Nutrient Database for Standard Reference [22]. The bowel habit logs evaluated tolerance and potential adverse events from study muffin consumption by asking a series of questions adapted from Lewis et al. [23], such as “describe the consistency of your stools,” and provided a space for comments. Participants were instructed to contact investigators immediately if abnormal changes in bowel habits, or the presence of blood or mucus, were observed. Muffin intake compliance was assessed through food intake journals.

### Subjective satiety measurements

Each VAS was 100 mm in length with questions at each end to indicate feelings of subjective satiety ranging from “not at all” to “very much or a lot”. The 7 questions were adapted from Flint et al. [24] and included “how hungry are you?”, “how satisfied do you feel?”, “how full do you feel?”, “how much do you think you can eat?”, “how pleasant would you find eating another mouthful of this food?” “would you like to eat something sweet?”, and “would you like to eat something fatty?”.

### Biomarkers

A phlebotomist drew approximately 12 mL of blood at each time point into EDTA vacutainers (BD Diagnostics, Franklin Lakes, NF, USA). The tubes were centrifuged at 3,200 rpm for 12 min at 4 °C. Plasma was immediately aliquoted into 1.5 mL Eppendorf microcentrifuge tubes and stored at −80 °C until analyzed for glucose, insulin, GLP-1, PYY, and leptin. Glucose was determined using a hexokinase colorimetric method (Stanbio Laboratory, Boerne, TX, USA) with an intra-assay coefficient of variation (CV) <6%. Total insulin (CV < 8%; Alpco, Salem, NH, USA), leptin (CV < 6%; Raybiotech, Norcross, GA, USA), and total PYY (PYY_(1–36)_ and PYY _(3–36);_ CV < 6%; EMD Millipore, Billerica, MA, USA) were measured using an enzyme-linked immunosorbent (ELISA) technique. Total GLP-1 (GLP-1_(7–36)_ and GLP-1 _(9–36)_; CV < 7%; Raybiotech, Norcross, GA, USA) concentrations were determined by an enzyme immunoassay protocol.

### Statistical analysis

To assess differences between and within the HAM-RS2 and control groups, change from mean baseline and final scores were calculated for anthropometric, body composition, satiety (after three questions were reverse coded), and biomarker concentrations from each individual blood collection time points (fasting, 15, 30, 60, and 120 min). Due to limited sample size and potential deviations from normality, primary analyses were conducted using nonparametric tests. The Wilcoxon Signed Ranked test compared differences within groups, while the Mann–Whitney *U* test examined between group differences. The total area under the curve (AUC) was calculated for all plasma biomarkers using the trapazoidal rule and was compared using the nonparametric tests described above. Pearson’s correlation coefficient examined associations between dependent outcomes. Data are presented as mean ± standard error of the mean (SEM), unless otherwise noted. SPSS version 19 (IBM Corporation, Armonk, NY, USA) and statistical significance was achieved with a *P*-value ≤ 0.05.

## Results

### Participants

**Fig. 1:**
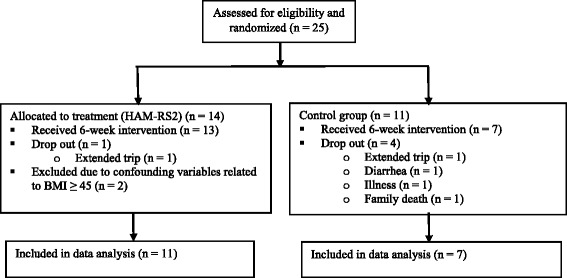
Consort Diagram

Twenty-five participants were enrolled in the study; however, only 18 (83% female) completed the protocol and were included in the data analysis (Fig. 1). Baseline characteristics of the participants who completed the study and were included in the data analysis did not differ between groups (Table 2).

**Table 2:** Baseline participant characteristics

Characteristic	HAM-RS2 (*n = 11*)	Control (*n = 7*)	*P* value^a^
Female, n (%)	9 (81.8)	6 (85.7)	
Age, years	31.0 ± 3.0	31.2 ± 4.2	0.973
Body mass index (kg/m^2^)	34.8 ± 1.5	30.6 ± 1.5	0.085
Waist circumference (cm)	99.1 ± 3.7	91.6 ± 3.3	0.151
Total body fat mass (kg)^b^	42.8 ± 3.1	34.6 ± 3.0	0.085
Total body lean mass (kg)^b^	47.3 ± 1.8	43.7 ± 2.3	0.179
Trunk total mass (kg)^b^	47.1 ± 3.6	43.9 ± 6.0	0.285
Trunk fat mass (kg)^b^	22.2 ± 2.1	19.8 ± 3.0	0.285
Trunk lean mass (kg)^b^	23.8 ± 1.8	23.1 ± 2.9	0.479
Android fat (kg)^b^	50.8 ± 2.1	50.1 ± 0.8	0.791
Gynoid fat (kg)^b^	52.2 ± 2.6	49.6 ± 1.0	0.285
Visceral adipose tissue (in^3^)^b^	76.8 ± 13.9	63.0 ± 18.1	0.417

Data presented as mean ± SEM except for qualitative values. All body composition measurements were obtained in triplicate after fasting for ≥8 h

^a^Between group differences determined by Mann–Whitney *U* test

^b^Measured by dual-energy x-ray absorptiometry (Lunar DPX NT model, GE Healthcare, Fairfield, CT, USA)

### Dietary intake and compliance

Energy and macronutrient (carbohydrate, protein, lipid) intake were similar between groups throughout the study (Table 3). Mean daily fiber intake significantly increased by 113% at midpoint in the HAM-RS2 group. At week 6, the mean fiber intake in the HAM-RS2 group increased 100% from baseline indicating fiber intake was sustained throughout the study. In contrast, the control group had a significant reduction in both fiber and carbohydrate intake from midpoint to the end of the study. Dietary fiber intake was considerably higher in the HAM-RS2 group at midpoint and the end of the study than the control. An unexplained reduction in dietary carbohydrate and fiber intake occurred from week 3 to week 6 in the control group. At baseline the HAM-RS2 group consumed a high fat diet averaging 95.8 ± 29.5 g/d (39.3% of total calories), which was non-significantly reduced to 82.1 ± 25.1 g/d (34.8% of total calories) at week 6. Based on data from the food intake journals, the HAM-RS2 and control groups consumed 94 and 98% of the study muffins, respectively, at midpoint suggesting high compliance. Compliance decreased to 85% in the HAM-RS2 group and 73% in the control group at the end of the study.

**Table 3:** Changes in mean macronutrient intake between HAM-RS2 and control groups^1,2^

Nutrient	HAM-RS2			Control			*P* value^**^
	Baseline	Week 3	Week 6	Baseline	Week 3	Week 6	
Calories	2190 ± 564	2193 ± 397	2123 ± 424	2091.1 ± 455.3	2046. ± 1337	1910.5 ± 648.7	0.256
Carbohydrate (g)	257.5 ± 84.8	283.0 ± 73.2	268.7 ± 62.6	294.3 ± 90.4	289.4 ± 173.7^a^	261.7 ± 84.0^a^	1.000
Protein (g)	82.7 ± 15.3	78.0 ± 13.2	78.4 ± 24.7	88.0 ± 11.1	74.8 ± 48.3	67.4 ± 32.7	0.462
Lipid (g)	95.8 ± 29.5	82.5 ± 20.7	82.1 ± 25.1	75.5 ± 23.9	70.1 ± 52.4	70.8 ± 27.0	0.462
Fiber (g)	21.5 ± 13.9^a,b^	45.7 ± 8.2^1*^	43.1 ± 6.7^b^	29.5 ± 14.5	30.2 ± 18.0^a*^	23.8 ± 6.8^a^	0.001

^1^Dietary analysis software used is United States Department of Agriculture (USDA) National Nutrient Database for Standard Reference (http://ndb.nal.usda.gov)

^2^Within group values with the same letter superscript (^a,b^) in the same row are statistically different (*P* < 0.05)

^*^Between group values are statistically different (*P* < 0.05) by Mann–Whitney *U* test

^**^Between group comparisons at week 6 analyzed by Mann–Whitney *U* test

### Tolerance to study muffins

In both groups, stool consistency was relatively stable throughout the study. No differences in reaching the bathroom in time, usage of toilet paper, or marks on undergarments occurred across groups at any time point. One participant in the HAM-RS2 group recorded the presence of blood or mucus in stool on two separate occasions; one prior to receiving the intervention and the other at midpoint. This participant was 89% compliant with muffin consumption at midpoint based on food records suggesting intake remained adequate. Overall, the HAM-RS2 and control muffins were well tolerated and few changes in bowel habit indicators were documented throughout the study.

### Subjective satiety

Mean overall satiety score did not differ within or between groups (*P* = 0.230). The mean score for the question “how full do you feel?” in the HAM-RS2 group increased from baseline to the end of the study and approached significance (*P* = 0.058) (data not shown).

### Body composition

Consuming muffins with HAM-RS2 did not significantly change body composition. A decrease (*P* = 0.043) in total trunk mass and a near significant decrease in lean trunk mass (*P* = 0.063) occurred in the control group (data not shown). At baseline the HAM-RS2 group had higher total leg mass (*P* = 0.011) than the control group, and the difference was sustained at the end of the intervention (*P* = 0.02) (data not shown).

### Biomarkers

**Fig. 2:**
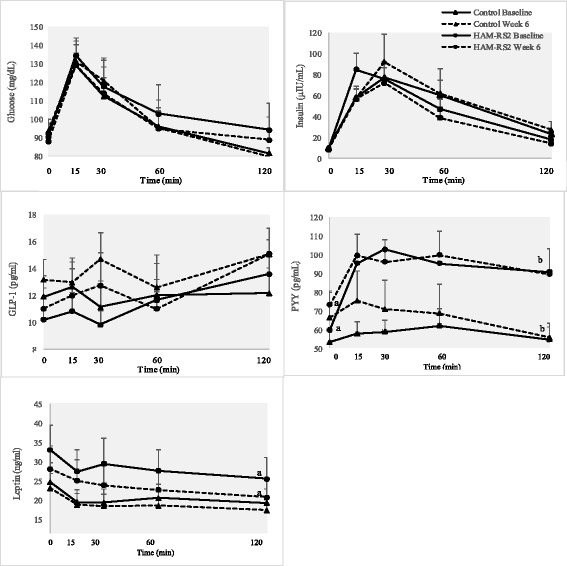
Change in biomarker concentrations from baseline to week 6 in the HAM-RS2 and control groups. This figure shows within-group comparisons from Wilcoxon Signed Ranked tests and between-group comparisons from Mann–Whitney *U* tests. ^a^Indicates significant within-group changes in biomarkers from baseline to week 6. Fasting PYY increased (*P* = 0.033) while leptin decreased (*P* = 0.028) 120 min after study foods were consumed in the HAM-RS2 group. A near-significant decrease (*P* = 0.062) in leptin also occurred 60 min after study foods were consumed in the HAM-RS2 group. ^b^Indicates significant difference at baseline between groups in 120 min postprandial PYY concentrations (*P* = 0.043)

Changes from baseline to week 6 in the AUC for the plasma biomarkers are shown in Table 4. At the end of the 6-week treatment, the AUC change from baseline was not significantly different between HAM-RS2 and control groups for any biomarker measures. However, a significant within-group decrease in both AUC glucose (*P* = 0.028) and AUC leptin (*P* = 0.022) was observed from baseline to week 6 in the HAM-RS2 group. There were no changes from baseline to the end of the intervention in the AUC for any biomarker in the control group. Within-group comparisons for biomarkers collected at each time point at the end of the intervention are shown in Fig. 2. Only one biomarker differed between groups when examining single blood collection time points. The 120 min postprandial concentration of PYY (*P* = 0.043) was higher in the HAM-RS2 group than the control group at baseline. Within-group differences in leptin and PYY were found from baseline to week 6 in the HAM-RS2 group. A decrease in postprandial leptin (*P* = 0.028) occurred at 120 min in the HAM-RS2 group. A within-group change in PYY was also observed in the HAM-RS2 group where fasting concentrations increased (*P* = 0.033) from baseline to the end of the intervention. Within the control group, no differences in the individual time points or AUC for any biomarker occurred.

**Table 4:** Mean AUC biomarker comparisons within and between groups

Biomarkers	HAM-RS2		Control		*P* value^1^
	Change from baseline to week 6	*P* value^2^	Change from baseline to week 6	*P* value^2^	
Glucose	−1588 ± 545	0.028	−790 ± 706	0.310	0.285
Insulin	−908 ± 941	0.333	518 ± 1197	1.000	0.425
GLP-1	120 ± 94	0.139	208 ± 127	0.176	0.791
PYY	438 ± 719	0.285	954 ± 1465	0.866	0.085
Leptin	−664 ± 235	0.022	−191 ± 230	0.398	0.425

Data presented as mean ± SEM

^1^Between group comparisons at week 6 by Mann–Whitney *U* test

^2^Within group comparisons from baseline to week 6 by Wilcoxon Signed-Ranked test

### Relationship between subjective satiety and biomarkers of satiety

Correlations between the mean AUC for each biomarker and the mean score for each VAS question were not found in either the control or HAM-RS2 group at the end of the intervention; however, correlations between body composition measurements and the AUC for several biomarkers were found. In the HAM-RS2 group, BMI (r = 0.655; *P* = 0.029), percent total fat (r = .889; *P* < 0.001), total trunk mass (r = 0.851; *P* = 0.001); trunk fat (r = 0.700; *P* = 0.017); trunk lean (r = 0.795; *P* = 0.003) were associated with the AUC glucose. The percent total body fat correlated (r = 0.652; *P* = 0.030) with AUC leptin. In the control group AUC glucose was associated with BMI (r = 0.814; *P* = 0.026), total fat (r = 0.801; *P* = 0.030), percent fat (r = 0.879; *P* = 0.009), and percent trunk fat (r = 0.772; *P* = 0.042). Total trunk lean mass correlated with the AUC insulin (r = 0.792; *P* = 0.034) in the control group. The AUC glucose was associated with the AUC insulin in both the HAM-RS2 (r = 0.710; *P* = 0.014) and control (r = 0.785; *P* = 0.036) groups.

## Discussion

Our primary aim was to examine changes in glucose homeostasis after consuming 30 g HAM-RS2 for 6 weeks in overweight adults. We also measured the plasma biomarkers (GLP-1, PYY, and leptin) and subjective satiety which could alter dietary intake and body composition. We found significant reductions in AUC glucose and AUC leptin in the HAM-RS2 group although differences between groups did not occur. In addition, a significant increase in fasting PYY occurred within the HAM-RS2 group after consuming the treatment muffins for 6 weeks. Interestingly, the favorable changes in biomarkers in the HAM-RS2 group did not elicit changes in overall mean subjective satiety score or body composition at the end of the intervention. Only one biomarker differed between groups throughout the duration of the study. Baseline PYY 120-min post-muffin intake was significantly higher in the HAM-RS2 group which may be attributed to initial HAM-RS2 fermentation. Increasing the duration of the. intervention or sample size may have produced additional between-group changes in biomarkers.

The decrease in AUC glucose in the HAM-RS2 group occurred under normoglycemic conditions and no change in overall mean carbohydrate intake suggesting other contributing mechanisms. One mechanism could be due to the SCFA produced from the fermentation of HAM-RS2 by bacteria in the lower GI tract. Butyrate and propionate are substrates for intestinal gluconeogenesis [25]. The newly synthesized glucose from the intestine reduces overall hepatic gluconeogenesis through portal vein sensors that contribute to overall blood glucose control [25].

Interestingly, HAM-RS2 lowered glucose AUC in the presence of a high-fat diet. At baseline habitual dietary fat intake in the HAM-RS2 group was 39.4% of total calories (~95 g per day). It is well established that diets high in fat consisting of large amounts of saturated and omega-6 polyunsaturated fatty acids and lower omega-3 polyunsaturated fatty acids contribute to chronic inflammation [26] and the development of chronic disease. Interestingly when dietary composition contains only 30.4% of calories from fat, HAM-RS2 can suppress inflammation and normalize glucose by mediating gluconeogenesis potential and altering hepatic fuel usage from lipids to carbohydrate [27]. Despite observing an improvement in AUC glucose in the HAM-RS2 group, we did not see changes in plasma insulin or insulin sensitivity (determined by Homeostasis Model Assessment estimates) that have been reported in human trials [13–15]. We did, however, observe a positive correlation between AUC glucose and AUC insulin in both groups.

A novel finding from our study is that AUC leptin significantly decreased from baseline to the end of the intervention in the HAM-RS2 group independent of changes in body composition. We also found a significant postprandial reduction at 120 min and near significant postprandial reductions at 30 (*P* = 0.074) and 60 min (*P* = 0.062) in leptin. Leptin is primarily produced by adipocytes and blood concentrations correlate with adipocyte size and percent body fat. One plausible mechanism could be enhanced fat oxidation which has been observed in healthy adults where postprandial fat oxidation increased 23% after 5.4% of dietary carbohydrate, but not 10.7%, was consumed acutely as HAM-RS2 [17]. Approximately 11% of mean daily carbohydrate intake was in the form of HAM-RS2 in our study; however, our population and study duration differed from Higgins et al. [17]. Another study found increased fat oxidation when resistant starch type 4 (RS4) plus whey protein was administered to healthy overweight and lean women [28]. Increased fat oxidation and resting energy expenditure also occurred in healthy lean males following consumption of 38 g RS4 in a mixed meal [29]. In addition, a fermentable cereal fiber reduced leptin by enhancing the gene expression of several enzymes involved in fat oxidation [30]. Similar to our findings, So et al. [31] reported lower leptin in addition to smaller adipocyte size in mice consuming HAM-RS2 compared to mice on a low resistant starch diet even though body composition did not differ between the groups. In contrast, no change in adipose leptin mRNA expression or plasma leptin concentrations occurred after a meal tolerance test in healthy males who consumed 30 g HAM-RS2 over 4 weeks [13]. The study by Robertson et al. examined healthy individuals with an average BMI of 23.7 kg/m^2^ which is much lower than our mean baseline BMI of 34.8 kg/m^2^, and our intervention was longer in duration. This suggests that duration of HAM-RS2 consumption and degree of adiposity may also be important in modulating leptin. Differences in adiposity (mean total body fat mass) between HAM-RS2 and control groups (*P* = 0.085) may explain why leptin did not differ between groups in our study, or that changes in leptin are more sensitive in individuals with higher fat mass.

The reduction in leptin after HAM-RS2 intake may benefit individuals with leptin resistance. Leptin resistance can develop from consuming high fat diets [12, 32] and sustained elevated leptin concentrations [33]. In our study, the HAM-RS2 group had a mean fasting leptin concentration of 33 ± 6 ng/mL. Fasting leptin concentrations of ≥15 ng/mL have been described as the cutoff value to predict insulin resistance [34]. Although we did not observe insulin resistance in the HAM-RS2 group, it is plausible that the study participants in our study were resistant to leptin due to high blood leptin concentrations and consumption of a high-fat diet at baseline. We observed significant reductions in leptin, but also a non-significant mean reduction (5.5%) in total calories from fat, but not overall total calories, in the HAM-RS2 group. This decrease in dietary fat is not likely responsible for the decrease in AUC leptin concentrations [35]. Similar to our results, leptin decreased with the addition of a fermentable soluble fiber in obese rats ingesting a high-fat diet [36]. Interestingly, the decrease in leptin in this study did not induce an orexigenic effect since overall caloric intake did not change and was similar to the control.

Our study also found an increase in total fasting PYY but not postprandial or AUC PYY in the HAM-RS2 group at the end of the study. The increase may be related to the carry-over fermentation effects of prior-day HAM-RS2 intake. Increased PYY has been observed with fermentable fiber consumption in animal studies [37]. PYY binds to the Y2 receptors of the arcuate nucleus to elicit a satiation response alongside increased energy expenditure [38]. Two isoforms of PYY exist: PYY_1–36_ and PYY_3–36_. PYY_1–36_ predominates under fasting conditions and has a lower affinity to the Y2 receptor. This may explain why we did not see a relationship between total PYY and satiety. At baseline, PYY was significantly higher (*P* = 0.043) in the HAM-RS2 group than control 120 min after the consumption of the study muffins and may be due to the initial fermentation of HAM-RS2 following intake. A similar increase in PYY occurred in healthy adults consuming resistant starch and whey protein 180 min after intake [28]. We did not observe improvements in GLP-1, which is consistent with several human studies examining HAM-RS2 intake in overweight adults for ≥4 weeks [39, 40].

This study has several limitations. First, only inactive concentrations of GLP-1 and total PYY (PYY_(1–36)_ + PYY_(3–36)_) were measured. We were not able to determine the exact PYY isoform which may have explained why subjective satiety did not change. Another limitation includes the lack of adding di-peptidyl peptidase-4 inhibitors (DPP-IV) following blood collection. DPP-IV rapidly degrades GLP-1, thus we were not able to measure the physiologically active form of GLP-1 [41]. Also, the participants consumed the study muffins at any time during the day. Consuming the muffins at one meal or equally distributed throughout the day could impact metabolic response. In addition, the participants consumed the study muffins prior to postprandial blood collection instead of an isocaloric mixed meal equal in macronutrient composition. Thus, the plasma biomarker response is reflective of study muffin consumption. However, a between group 120-min improvement in PYY was observed in the HAM-RS2 group indicating fermentation can upregulate this satiety peptide. Lastly, a significant reduction in daily fiber intake from midpoint to week 6 occurred in the control group indicating the control muffins replaced other high-fiber foods in the diet.

## Conclusion

In conclusion, daily consumption of 30 g of HAM-RS2 in muffins over 6 weeks can decrease leptin concentrations, assist with blood glucose homeostasis, and improve fasting PYY in healthy overweight adults. These findings occurred without changes in total caloric intake or body composition. Adding HAM-RS2 to the diet can improve fiber intake to enhance overall diet quality. However, strong conclusions cannot be made due to small sample size and between group differences in biomarkers were not observed at the end of the intervention. The mechanisms associated with biomarker changes in the HAM-RS2 group are likely related to the fermentation of HAM-RS2 by gut microbiota, but additional research is needed to determine the type, amount, and duration of resistant starch that would provide the most advantageous physiological results.

## Abbreviations

AUC: Area under the curve
BMI: Body mass index
DXA: Dual-energy X-ray absorptiometry
GI: Gastrointestinal
GLP-1: Glucagon-like peptide-1
HAM-RS2: High-amylose maize resistant starch type 2
PYY: Peptide YY
RDN: Registered Dietitian Nutritionist
SCFA: Short chain fatty acids
SEM: Standard error of the mean
VAS: Visual analogue scale
